# Pull-in suture: a novel reconstruction technique for tendon avulsion injury at the musculotendinous junction associated with forearm open fracture

**DOI:** 10.1080/23320885.2022.2054812

**Published:** 2022-04-06

**Authors:** Yuta Izawa, Yoshihiko Tsuchida, Hiroko Murakami, Tetsuya Shirakawa, Masahiro Nishida, Kentaro Futamura

**Affiliations:** a Department of Orthopedic Trauma Center, Sapporo Higashi Tokushukai Hospital, Sapporo, Japan; b Department of Trauma Center, Shonan Kamakura General Hospital, Kanagawa, Japan

**Keywords:** Avulsion injuries of the tendons at the musculotendinous junction, forearm open fractures, pull-in suture, tendon transfer

## Abstract

We present three cases of strong one-staged tendon reconstruction for musculotendinous junction avulsion tendon injuries, and called it a ‘pull-in suture’. The clinical outcomes of this method are comparable to those of tendon transfer; it is an effective reconstruction method that should be considered as an initial treatment procedure.

## Introduction

Avulsion injuries of the tendons at the musculotendinous junction associated with forearm open fractures present as challenging cases for trauma surgeons and hand surgeons [1], although they are rare injuries [2–5]. Patients with tendon avulsions at the musculotendinous junction risk being significantly disabled; thus, it is important to establish a surgical procedure that will optimize the outcomes in such cases. In cases of complete tendon rupture at the musculotendinous junction, direct repair is often impossible because the local pathology makes the muscle end unsuitable for repair [6,7]. The outcome of one-stage reconstruction using end-to-end repair is considered poor because of the persisting limitation in the range of motion of the finger. It has been pointed out that the cause is myostatic contracture, which causes contracture of the wrist joints and fingers due to a decrease in the sliding distance of the muscles [8,9]. Therefore, side-to-side repair and tendon transfer have been recommended as treatments for avulsion injuries [10,11]. We considered that the poor outcomes associated with conventional one-stage end-to-end repair were because the procedure cannot produce sutures that can withstand range of motion training while maintaining the appropriate tension. Therefore, we performed strong one-stage tendon repair procedures in which a suture was fixed to the intact fascia on the proximal side, and called it a ‘pull-in suture’[12]. We report the postoperative outcomes of three such cases where pull-in sutures were performed for avulsion injuries of tendons at the musculotendinous junction associated with forearm open fracture.

## Patients and methods

**Figure 1.**
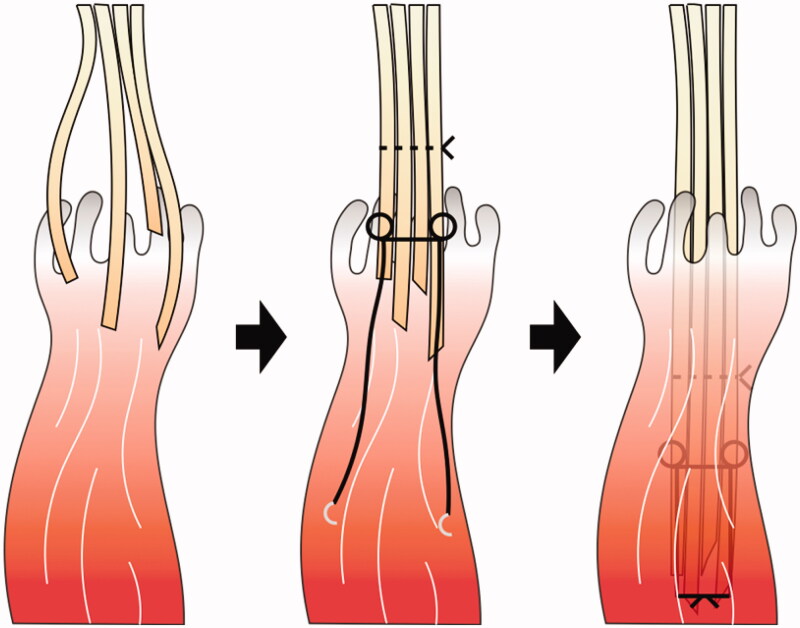
Schematic diagram of a pull-in suture.

Three cases of avulsion injuries of tendons at the musculotendinous junction associated with forearm open fracture were included in this study. In all cases, irrigation, debridement and temporary fixation for the fractures were performed on the day of injury. Definitive internal fixation, tendon repair, and soft tissue reconstruction were performed as required within a few days. The following procedure for one-staged reconstruction was performed for tendon repair, defined as ‘pull-in sutures’ for avulsion tendon injuries at the musculotendinous junction (Figure 1): first, the damaged tendon was identified, and the muscle tissue attached to the distal stump of the tendon was debrided. The proximal muscle tissue was debrided only in the crushed and contaminated areas. Tendons derived from the same muscle were bundled using a nylon thread according to the Kessler method. The bundled tendons were pulled into the proximal muscle body from which they originated, and the nylon thread was sutured to the intact fascia. After the surgery, strong tension to the tendons was avoided as much as possible, and only passive tenodesis-like motion conducted by the rehabilitation staff was allowed for 3 weeks. Active and passive range of motion training was initiated 3 weeks postoperatively. Tendon transfer was considered if good range of motion could not be obtained even after continuous finger flexion and extension range of motion training for 3 months after the initial stage surgery. At the final follow-up, the total active motion (TAM) of the thumb, TAM of the index finger to the little finger [13], the range of motion of the wrist joint, and the Disabilities of the Arm, Shoulder and Hand (DASH) score were measured [14].

## Cases

### Case 1

**Figure 2.**
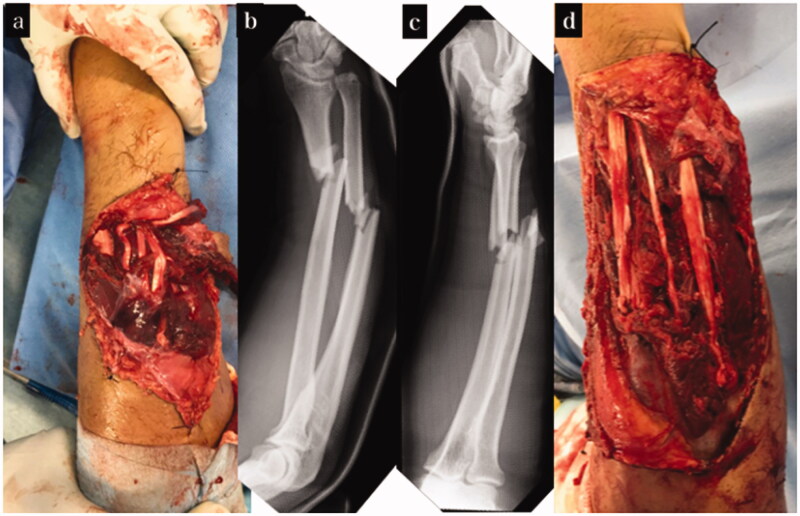
Treatment course of case 1. (a) Appearance on arrival at our hospital. (b,c) X-ray on arrival at our hospital. (d) Appearance after tendon reconstruction using pull-in sutures.

**Figure 3.**
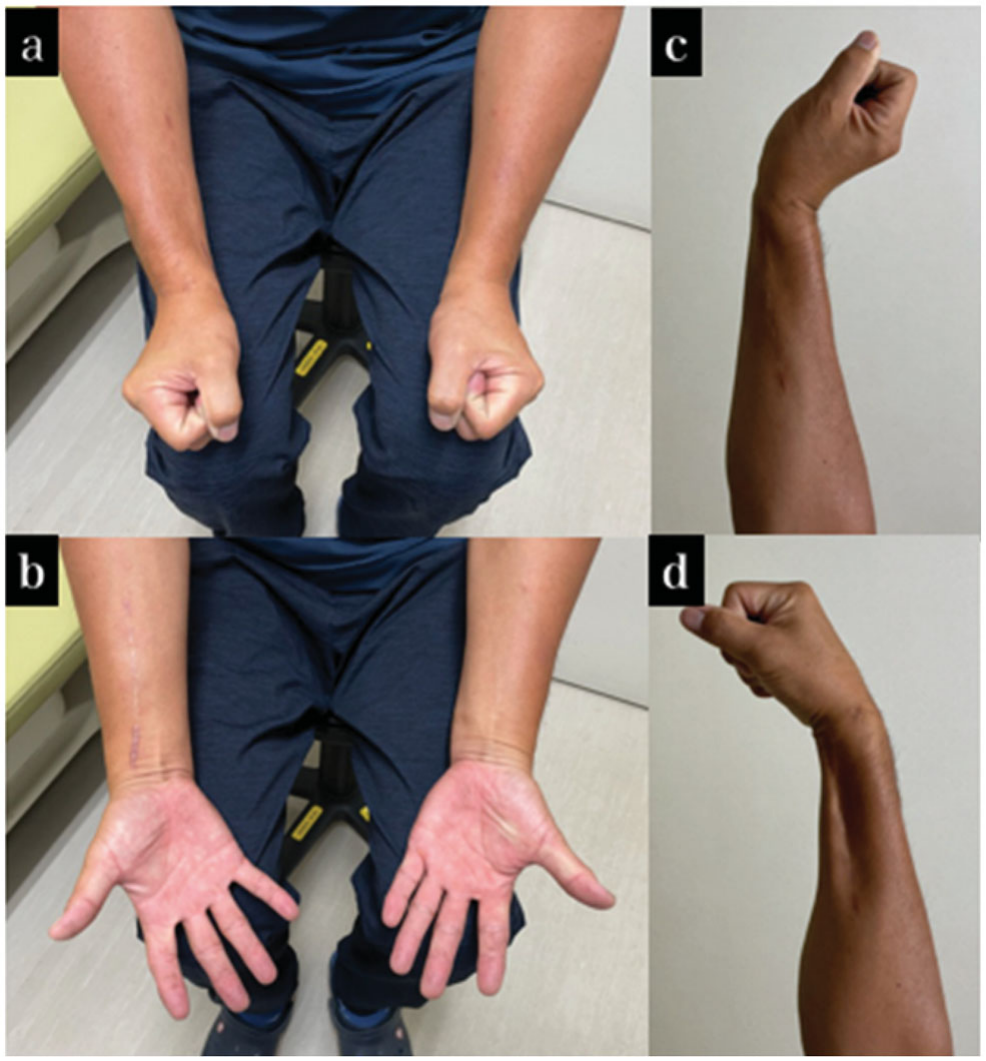
Final appearance and range of motion of case 1. (a–d) Appearance and range of motion at 1 year after the injury.

A male patient aged 49 years sustained an injury in the right upper extremity when it was caught in an agricultural machine. We diagnosed the patient with right humeral shaft fracture, right forearm open fracture with a Gustilo–Anderson classification of type 3 A, multiple rib fractures, liver injury, and right lung contusion, with associated right radial nerve insufficiency paralysis. On the day of the injury, irrigation and debridement of the open wound and wire fixation for forearm fractures were performed. When the open wound was extended and damaged structure was confirmed, the damage was localized to the dorsal side of the forearm, and no neurovascular injury was observed. The extensor pollicis longus (EPL), extensor digitorum (EDC), extensor digitorum minimi (EDM), and extensor carpi ulnaris (ECU) tendons were pulled out at the musculotendinous junction and completely torn. Although extensor carpi radialis longus (ECRL) and extensor carpi radialis brevis (ECRB) were intact, it was difficult to extend the wrist joint. Since there was a decrease in sensation in the radial nerve region, it was judged to be due to radial nerve insufficiency paralysis associated with a humeral shaft fracture. The open wound was closed. Osteosynthesis was performed for humeral shaft fracture on the 3rd day of injury, and the EPL, EDC, EDM, and ECU were reconstructed using pull-in sutures. On the 10th day of injury, the skin necrosis was debrided, and the wire that had been inserted in the ulnar was replaced with an intramedullary nail. After the tendons repair surgery, strong tension to the tendons was avoided as much as possible, and only passive tenodesis-like motion was allowed for 3 weeks. Active and passive range of motion training was initiated 3 weeks postoperatively. Since there was no flexor tendon injury, range of motion training of the finger and wrist was initiated with the goal being two-stage tendon transfer. Three months after the injury, we planned to perform osteosynthesis for the radial shaft fracture using a plate, along with extensor tendon reconstruction with tendon transfer if required; however, we did not perform the tendon transfer because the range of motion of the fingers had improved (Figure 2). One year after the injury, the TAM of the thumb was good and the TAM of the index finger to the little finger was good to excellent (Table 1). The wrist joint active palmar flexion and active dorsiflexion were 55° and 65°, respectively, and the DASH score was 10.8. The radial nerve palsy had recovered completely. Although mild restriction in wrist flexion remained, the patient resumed his job in agriculture (Figure 3).

**Table 1.** TAM at the final follow-up of each case.

Case	Finger	TAM (°)	%TAM (%)	Evaluation
1	Thumb	98	86.0	Good
	Index	213	87.0	Good
	Middle	228	91.2	Excellent
	Ring	206	85.1	Good
	Little	210	89.0	Good
2	Thumb	102	86.4	Good
	Index	216	91.5	Excellent
	Middle	220	94.0	Excellent
	Ring	220	91.6	Excellent
	Little	206	85.1	Good
3	Thumb	58	26.7	Poor
	Index	262	89.2	Good
	Middle	258	86	Good
	Ring	248	84.4	Good
	Index	238	95.2	Excellent

### Case 2

**Figure 4.**
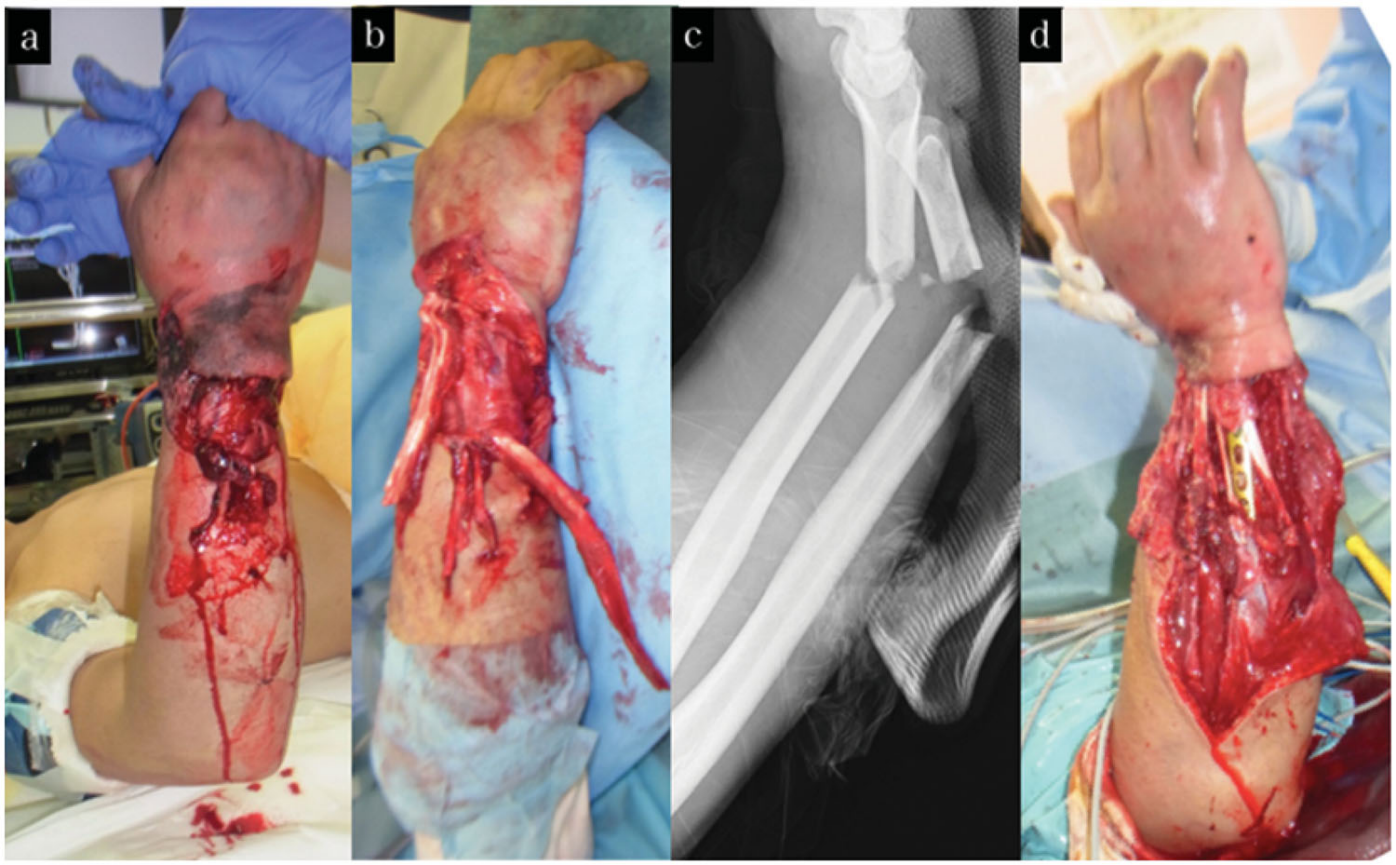
Treatment course of case 2. (a,b) Appearance on arrival at our hospital. (c) X-ray on arrival at our hospital. (d) Appearance after tendon reconstruction using pull in suture and osteosynthesis.

**Figure 5.**
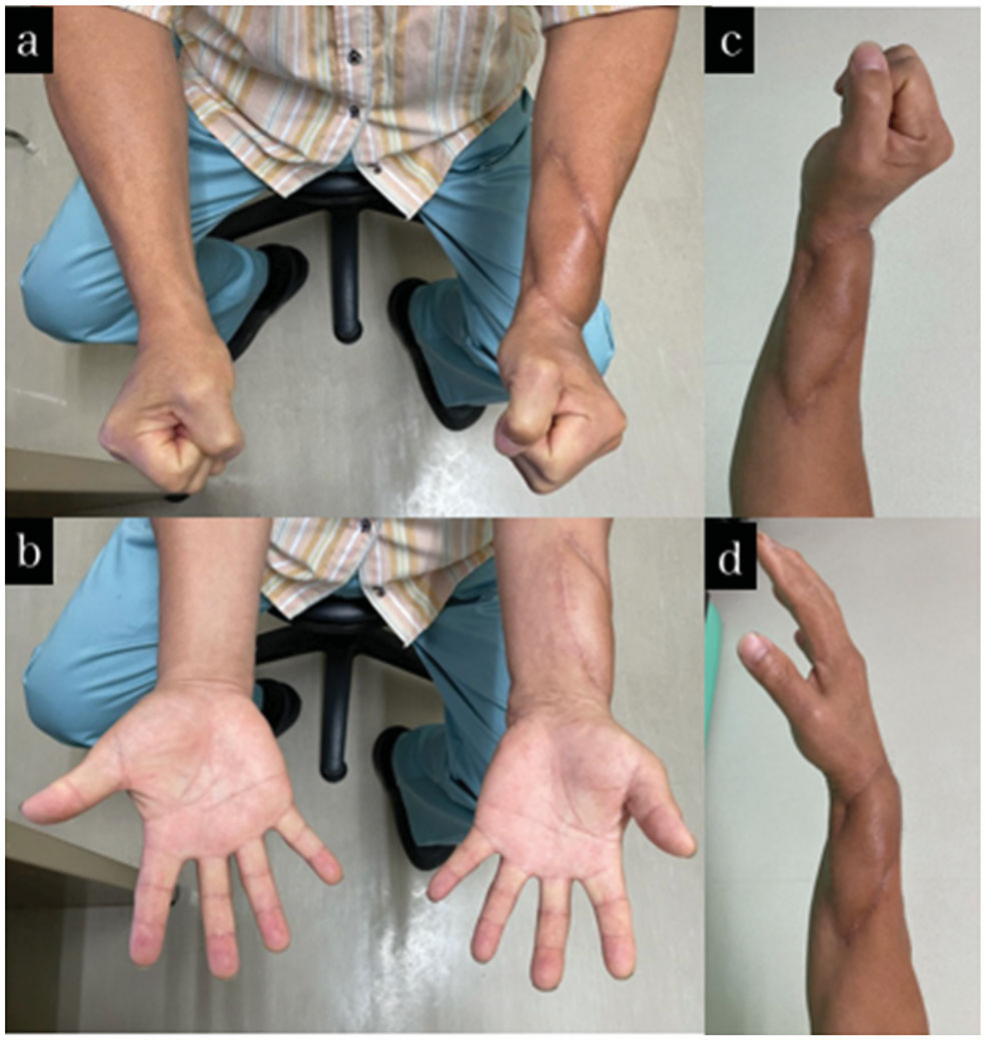
Final appearance and range of motion of case 2. (a–d) Appearance and range of motion at 1 year after the injury.

A male patient aged 62 years, was injured when his right upper extremity was caught in a machine in the factory. He was diagnosed with a Gustilo–Anderson type 3B right forearm open fracture with a skin defect of 12 × 5 cm. Irrigation and debridement of the open wound were performed on the day of the injury. There was no neurovascular injury to the forearm. The EPL, EDC, extensor indicis proprius, EDM, and ECU tendons were pulled out at the musculotendinous junction and completely torn. On the second and third days after injury, osteosynthesis was performed for the forearm shaft fractures with a plate, and all of the ruptured tendons were reconstructed using pull-in sutures and soft tissue reconstruction with an anterolateral thigh flap. After the tendons repair surgery, strong tension to the tendons was avoided as much as possible, and only passive tenodesis-like motion was allowed for 3 weeks. Active and passive range of motion training was initiated 3 weeks postoperatively. The flap survived without any complications, and debulking surgery was performed 9 months after the injury (Figure 4). Two years after the injury, the TAM of the thumb and little finger was good, and the TAM of the index finger to the ring finger was excellent (Table 1). The wrist joint active palmar flexion and active dorsiflexion were 35° and 45°, respectively, and the DASH score was 21.6. Although mild restrictions in the skilled movement remained, the patient resumed his job as a sheet metal worker with no complaints (Figure 5).

### Case 3

**Figure 6.**
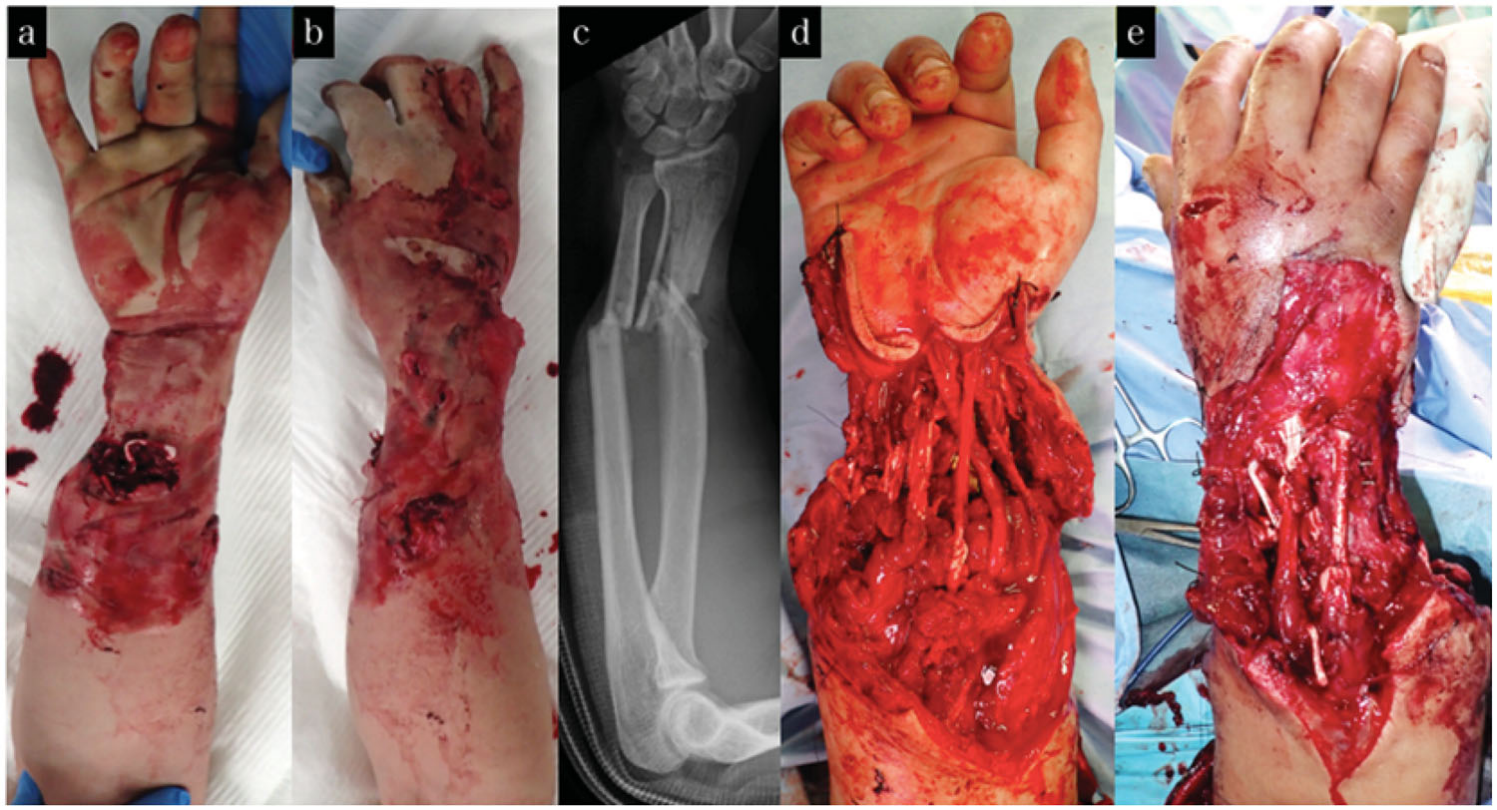
Treatment course of case 3. (a,b) Appearance on arrival at our hospital. (c) X-ray on arrival at our hospital. (d,e) Appearance after tendon reconstruction using pull in suture.

**Figure 7.**
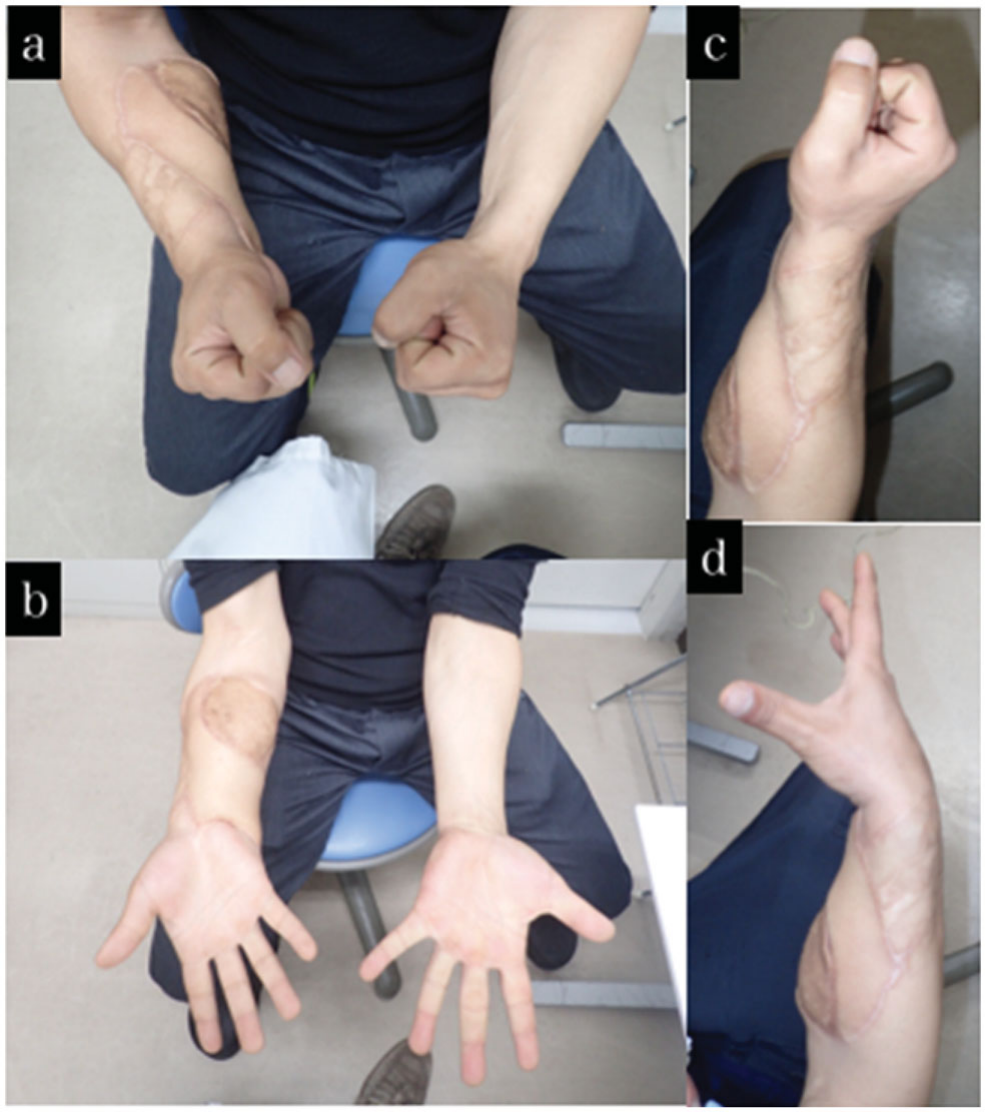
Final appearance and range of motion of case 3. (a–d) Appearance and range of motion at one year after the injury.

A male patient aged 21 years was injured when his right upper extremity was caught in a machine at a factory. This was a forearm insufficiency amputation case in which only some skin and muscle tendons of the right hand remained. On the day of the injury, revascularization, irrigation, and debridement of the open wound were performed. The only remaining tendons were the flexor policis longus (FPL) and the flexor carpi ulnaris (FCU); all other tendons were pulled out at the musculotendinous junction and completely torn. On the third day of injury, osteosynthesis was performed for the forearm shaft fracture, and the EPL, EDC, ECU, flexor carpi radialis, and flexor digitorum profundus tendons were reconstructed using a pull-in suture. Subsequently, soft tissue reconstruction was performed using a free latissimus dorsi myocutaneous flap (Figure 6). After the tendons repair surgery, strong tension to the tendons was avoided as much as possible, and only passive tenodesis-like motion was allowed for 3 weeks. Active and passive range of motion training was initiated 3 weeks postoperatively. Two years after the injury, the TAM of the thumb was poor and the TAM of the index finger to the little finger was good to excellent (Table 1). The wrist joint active palmar flexion and active dorsiflexion were 65° and 60°, respectively, and the DASH score was 7.5. He returned to his former job without complaints (Figure 7).

## Discussion

In cases of complete tendon rupture at the musculotendinous junction, direct repair is often impossible. The local pathology makes the muscle end unsuitable for repair [6,7]. Although one-stage end-to-end repair, end-to-side repair, side-to-side repair, and other methods have been reported as reconstruction techniques, the outcome of the one-stage reconstruction using end-to-end repair has been regarded as poor because limitation of the range of motion of the finger often persists. Reportedly, the cause of this is myostatic contracture, which causes contracture of wrist joints and fingers due to a decrease in the sliding distance of muscles [8,9]. It is recommended that the avulsed extensor and flexor tendon repair be a two-stage tendon transfer by selecting a force source after sufficient prevention of joint contracture. Collins et al. conducted a systematic review on the treatment of avulsion injuries of tendons at the musculotendinous junction and proposed treatment algorithms according to each type and injury site [1]. They recommended reattachment or tendon transfer for flexor tendon injuries without amputation and tendon transfer or side-to-side repair for extensor tendon injuries without amputation. However, our patients had multiple tendon avulsion injuries with open forearm fractures, and it would be difficult to apply the recommendations by Collins et al. in these cases.

While there are some reports that tendon transfer for tendon rupture at the musculotendinous junction has achieved the same range of motion as the uninjured side, there are also many reports that the range of motion has decreased by about 30 degrees compared to the uninjured side [8]. The three patients in this study had postoperative outcomes comparable to those reported for tendon transfer. Our pull-in suture method differs from that for conventional end-to-end or buried sutures, which have been reported to have poor outcomes, in that the suture on the proximal side is applied to the intact fascial part that can be firmly fixed. Avoiding damaged fascia and proximal suturing can prevent re-rupture of the muscle tendon. Givissis et al. reported a tendon reconstruction method similar to that of the pull-in suture [15]. They reported good postoperative outcomes by encapsulating the tendon stump in the proximal muscle body for an FPL avulsion injury at the musculotendinous junction. Unlike simple sutures, encapsulation is considered to have achieved the same good outcomes as the pull-in suture in terms of the repaired tendon achieving appropriate tension and strength.

In the three patients in this study, the final TAM from the index finger to the little finger was good to excellent, and we believe that the aforementioned extension contracture due to myostatic contracture did not occur. Finger extension contracture can occur when the muscle body is severely damaged or defective, but pull-in sutures for patients with an adequately preserved muscle body can help withstand range of motion training while maintaining appropriate tension. Therefore, extension contracture due to myostatic contracture is unlikely to occur. Thus, the criteria for proper use of pull-in suture and tendon transfer can be clarified by examining the degree of muscle injury that can cause extension contracture due to myostatic contracture.

The strengths of the pull-in suture method lie in the potential to obtain a finger range of motion equivalent to that of tendon transfer without sacrificing the transfer tendon, in that the tendon can be reconstructed prior to soft tissue reconstruction, and in that it precludes complicated procedures such as tendon transfer. The disadvantage of pull-in sutures is that wrist and finger extension contracture due to myostatic contracture can occur in cases of severe muscle body damage. In addition, it may blunt the decision as to whether or not to perform two-staged tendon transfer and may lead to a final decline in functional performance. For cases of extensor tendon avulsion injury accompanied by severe soft tissue defects, pull-in sutures and soft tissue reconstruction should be performed after considering the residual structures and the degree of muscle body damage. If the improvement in finger range of motion following pull-in sutures is poor, two-stage tendon transfer should be considered. In patients with tendon avulsion injuries associated with complete or incomplete forearm amputation, pull-in sutures should be performed as part of the replantation procedure because tendon transfer cannot be performed in such cases.

## Conclusion

We reported the postoperative outcomes of three cases where pull-in sutures were used for avulsion injuries of the tendons at the musculotendinous junction associated with forearm open fracture. The clinical outcomes of the pull-in suture method are comparable to those of tendon transfer without sacrificing the transfer tendon, and it is an effective reconstruction method that should be considered as an initial treatment procedure for tendon avulsion injuries.
